# Identification of the Tolfenamic Acid Binding Pocket in PrbP from *Liberibacter asiaticus*

**DOI:** 10.3389/fmicb.2017.01591

**Published:** 2017-08-23

**Authors:** Lei Pan, Christopher L. Gardner, Fernando A. Pagliai, Claudio F. Gonzalez, Graciela L. Lorca

**Affiliations:** Department of Microbiology and Cell Science, Genetics Institute, Institute of Food and Agricultural Science, University of Florida Gainesville, FL, United States

**Keywords:** transcriptional accessory protein, *Liberibacter asiaticus*, tolfenamic acid, antimicrobial, citrus, binding pocket

## Abstract

In *Liberibacter asiaticus*, PrbP is an important transcriptional accessory protein that was found to regulate gene expression through interactions with the RNA polymerase β-subunit and a specific sequence on the promoter region. It was found that inactivation of PrbP, using the inhibitor tolfenamic acid, resulted in a significant decrease in the overall transcriptional activity of *L. asiaticus*, and the suppression of *L. asiaticus* infection in HLB symptomatic citrus seedlings. The molecular interactions between PrbP and tolfenamic acid, however, were yet to be elucidated. In this study, we modeled the structure of PrbP and identified a ligand binding pocket, TaP, located at the interface of the predicted RNA polymerase interaction domain (N-terminus) and the DNA binding domain (C-terminus). The molecular interactions of PrbP with tolfenamic acid were predicted using *in silico* docking. Site-directed mutagenesis of specific amino acids was followed by electrophoresis mobility shift assays and *in vitro* transcription assays, where residues N107, G109, and E148 were identified as the primary amino acids involved in interactions with tolfenamic acid. These results provide insight into the binding mechanism of PrbP to a small inhibitory molecule, and a starting scaffold for the identification and development of therapeutics targeting PrbP and other homologs in the CarD_CdnL_TRCF family.

## Introduction

Huanglongbing (HLB) or citrus greening, is a devastating disease threatening the citrus industry in Asia, Africa, and America (Graça, 1991; da Graça and Korsten, 2004; Bové, 2006; Gottwald, 2010; Wang and Trivedi, 2013). The causative agent is the phloem-limited bacteria *Liberibacter asiaticus, L. africanus, and L. americanus* (Bové, 2006; Gottwald, 2010; Wang and Trivedi, 2013). Although, it was first documented in Asia over a century ago (Graça, 1991; da Graça and Korsten, 2004; Bové, 2006), HLB was not widely recognized until its emergence in the United States between 2004 and 2005 (Bové, 2006). In Florida, the causing agent of HLB has been identified as *L. asiaticus* (Sagaram et al., 2009; Tyler et al., 2009). Despite efforts of the research community, the laboratory culture conditions of *L. asiaticus* remain elusive (Davis et al., 2008; Sechler et al., 2009; Parker et al., 2014), which has severely impeded the understanding of the physiology of *L. asiaticus*, and the development of an effective treatment for HLB.

In 2009, the first complete genome sequence of *L. asiaticus* was published (Duan et al., 2009). *L. asiaticus* has a relatively small genome (1.23 Mbps) that lacks genes encoding traditional pathogenicity determinants (Pagliai et al., 2014). Another salient feature revealed by genomic analysis is that only 2% of the *L. asiaticus* genome is predicted to encode transcription factors (Pagliai et al., 2014), significantly lower than many other common model organisms (e.g., 6.4% in *Escherichia coli*, 9.4% in *Pseudomonas aeruginosa*, 6% in *Sinorhizobium meliloti*; Stover et al., 2000; Pagliai et al., 2014). Interestingly, half of the predicted transcription factors in *L. asiaticus* were up-regulated *in planta* when compared to their expression in the psyllid vector (Yan et al., 2013). These data suggest that *L. asiaticus* may be adapted to each of its hosts (psyllid and citrus) by sensing specific host-derived signals which are sensed by specific transcription factors. These observations led to our hypothesis that the inactivation of critical transcriptional regulators in *L. asiaticus* may cause pleiotropic effects that result in compromised viability or reduced persistence of the pathogen during citrus infection.

PrbP is a member of CarD_CdnL_TRCF family, a distinct group of proteins that regulate the transcription machinery through interactions with the RNA polymerase (Padmanabhan, 2001; Penalver-Mellado et al., 2006; Stallings et al., 2009; Garcia-Moreno et al., 2010; Weiss et al., 2012; Gallego-García et al., 2014; Bernal-Bernal et al., 2015; Gardner et al., 2016). *Myxococcus xanthus* encodes for two members of this family, *carD* and *cdnL. Myxococcus* CarD, in complex with the zinc-binding protein CarG, was found to regulate various processes including oxidative stress response (induced by blue light) and vegetative growth under starvation conditions (Nicolas et al., 1994; Galbis-Martinez, 2004; Penalver-Mellado et al., 2006; Galbis-Martinez et al., 2008; Elías-Arnanz et al., 2010; Abellón-Ruiz et al., 2014). CdnL is a smaller protein (164 aa) that is essential for *M. xanthus* viability, and is homologous to the *Myxococcus* CarD N-terminal domain (Garcia-Moreno et al., 2010; Gallego-García et al., 2014). In *Mycobacterium*, CarD (a sequence homolog of *M. xanthus* CdnL) was also found to be essential for viability (Stallings et al., 2009), as well as a critical component for antibiotic resistance and multiple stress responses (Stallings et al., 2009; Weiss et al., 2012; Garner et al., 2014). CarD is also a critical component in pathogenesis and persistence during *M. tuberculosis* infection in mice (Stallings et al., 2009; Weiss et al., 2012; Garner et al., 2014). Furthermore, *Mycobacterium* CarD plays a general role in global regulation by binding non-specifically to the promoter region of hundreds of genes, and stabilizing the RNA polymerase open complex (Stallings et al., 2009; Gulten and Sacchettini, 2013; Landick et al., 2014; Rammohan et al., 2015).

**Figure 1:**
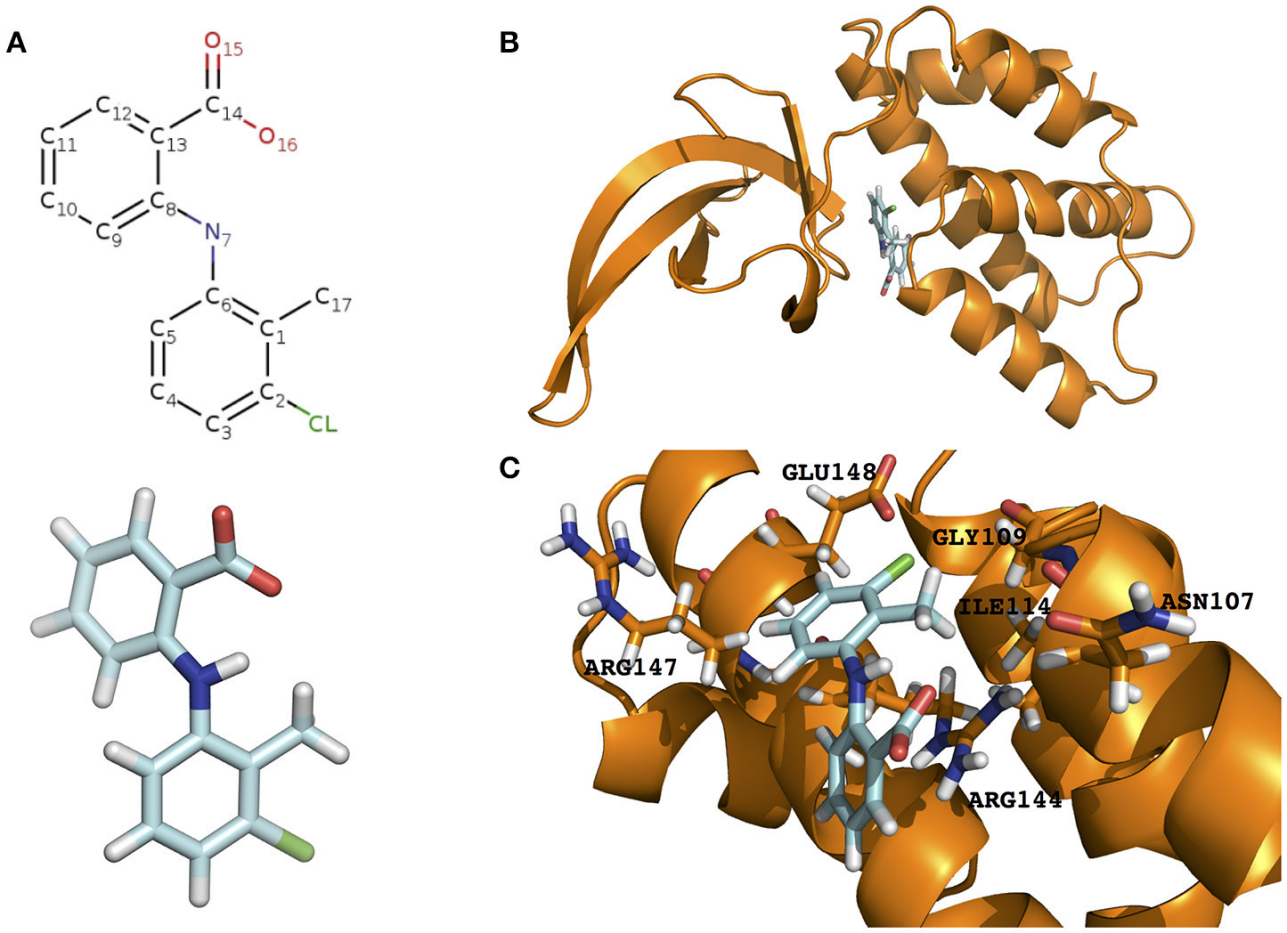
Prediction of the tolfenamic acid binding pocket in the model PrbP-MI and the amino acids involved in PrbP/ligand interactions. **(A)** Structure of tolfenamic acid (PDB#TLF). **(B)** The PrbP-MI model using *T. thermophilus* CarD co-crystallized with RNA polymerase β-1 lobe (PDB# 4XAX, Bae et al., 2015) as template. Tolfenamic acid is shown docked in TaP located at the interface of RID and CTD. **(C)** Close-up view of the predicted interacting amino acids in PrbP-MI model. Orange, cartoon representation of the PrbP-MI; blue sticks, structure of tolfenamic acid; orange sticks, predicted interacting amino acids in PrbP.

Contrary to other PrbP homologs, in *L. asiaticus*, PrbP binds specifically to the promoter regions of the *rplK* gene, and the 16S rRNA gene (Gulten and Sacchettini, 2013; Landick et al., 2014; Gardner et al., 2016). Using high-throughput screening of chemical libraries, we identified a small molecule, tolfenamic acid, that binds to PrbP (Figure 1A). Tolfenamic acid is a generic NSAID drug that is used to treat migraines in the UK, Latin America, and European countries (Hakkarainen et al., 1979; Hansen, 1994). *In vitro*, tolfenamic acid can effectively reduce PrbP interactions with the promoter region of the *rplK* gene (Gardner et al., 2016). *In vivo* tests of this potential PrbP inhibitor, using the surrogate strain *L. crescens*, suggest PrbP interactions with DNA may be physiologically important, as the addition of tolfenamic acid in culture was able to inhibit *L. crescens* growth (Gardner et al., 2016). Foliar application of tolfenamic acid on HLB-symptomatic leaves and sweet orange (*Citrus sinensis*) seedlings significantly decreased the overall transcriptional activity of *L. asiaticus*, and inhibited the infection progression (Gardner et al., 2016). These findings support that tolfenamic acid could be a promising antimicrobial targeting PrbP for therapeutic treatment of HLB, however, the molecular interactions between PrbP and tolfenamic acid are yet to be elucidated.

In this study, we focused on the identification and characterization of the tolfenamic acid binding pocket (TaP, Tolfenamic acid Pocket) in PrbP. A structural model of PrbP was constructed based on crystalized homologs, and *in silico* docking was used to predict the amino acids that mediate interactions with tolfenamic acid. We then used electrophoresis mobility shift assays (EMSA) and *in vitro* transcription assays to validate the *in silico* predictions. In summary, this study provides insights into the inhibition mechanism for PrbP function that could be used to develop therapeutics against members of the CarD_CdnL_TRCF protein family.

## Materials and methods

### Bioinformatics

The PrbP sequence was retrieved from the NCBI protein database (ACT56890). The structural modeling was performed under automated mode using SWISS-MODEL (Grosdidier et al., 2011). The results of the modeling are provided on Supplementary Table 1. The 3D structure of tolfenamic acid was extracted from ZINC database (ZINC00002188). The *in silico* docking was performed using SwissDock (Grosdidier et al., 2011) with the following parameters: PASSIVEFLEXIBILITYDISTANCE: 0.0, WANTEDCONFS: 5,000, NBFACTSEVAL: 5,000, NBSEEDS: 250, SDSTEPS: 100, ABNRSTEPS: 250, CLUSTERINGRADIUS: 2.0, MAXCLUSTERSIZE: 8. Prediction of the ligand interactions with PrbP was done by the Molecular Docking server (Bikadi and Hazai, 2009) using default parameters. The docking hits obtained from SwissDock were manually examined and the ligand orientation that matched to the most favorable prediction of ligand/protein interactions predicted in Molecular Docking server was chosen.

To perform multiple sequence alignments, PrbP homolog sequences were retrieved from the NCBI protein database, and alignments were performed using Clustal Omega (Sievers et al., 2011) with default parameters. To perform structural alignments with PrbP homologs that lack 3D-structures, the amino acid sequences were obtained from NCBI and modeled as described above. All available 3D-structures were obtained from Protein Data Bank (Berman et al., 2000). PyMol was used to visualize data and perform structural alignments.

### Strains and DNA manipulation

*Escherichia coli* DH5α and *E. coli* JM110 cells were used to maintain and replicate all plasmids. *E. coli* BL21 (DE3) (Novagen) was used for over-expression and further purification of recombinant proteins. The cells were grown in Luria-Bertani (LB) medium, at 37°C, in a rotary shaker (250 rpm). The culture media was supplemented with ampicillin (100 μg/ml), kanamycin (30 μg/ml) and/or chloramphenicol (34 μg/ml) when necessary. All antibiotics and chemicals were purchased from Sigma-Aldrich.

Chromosomal DNA was isolated with Qiagen DNAeasy Kit and plasmid extractions were performed with QIAprep Spin Miniprep Kit (Qiagen). Standard molecular protocols described in *Molecular Cloning* (Sambrook et al., 2001) were used to perform polymerase chain reactions (PCR), restrictive enzyme digestion, construction of recombinant DNA molecules, and cell transformations. Q5 high fidelity DNA Polymerase (NEB) was used for PCR reactions. PrbP site-directed mutagenesis was performed using the QuikChange Site-directed Mutagenesis kit (Agilent Technologies) following the manufacturer's protocol. All predicted residues were mutated to alanine using the plasmid p15TV-L carrying *prbP* as the template.

*L. asiaticus* RNA polymerase subunits β and β′ were cloned into plasmid pACYCDuet-1. Subunits α and ω were cloned into plasmid pRSFDuet-1. Sigma factor 70 was cloned into plasmid p15TV-L. The N-terminal 6X His-tag was fused to subunits β′, ω and sigma factor 70. Reproduction and manipulation of plasmids was done using *E. coli* DH5α or, in the case of plasmid pACYCDuet-1 and its derivatives, in *E. coli* JM110. All of the plasmids containing subunits of the RNA polymerase were co-transformed into *E. coli* BL21 (DE3), and the proteins were simultaneously over-expressed with IPTG for *in vivo* reconstruction of the *L. asiaticus* RNA polymerase holoenzyme.

All cloned DNA fragments described herein were verified by DNA sequencing. All of the strains, plasmids and primers are listed in Tables 1, 2.

**Table 1:** Strains and Plasmids used in this study.

**Name**	**Genotype/Description**	**Origin/Reference**
**BACTERIAL STRAINS**		
*Escherichia coli* DH5α	F^−^Φ80*lac*ZΔM15 Δ(*lac*ZYA-*arg*F) U169 *rec*A1 *end*A1 *hsd*R17 (${r_{K}}^{-}$, ${m_{K}}^{+}$) *pho*A *sup*E44 λ– *thi*-1 *gyr*A96 *rel*A1	Invitrogen
*E. coli* BL21 (DE3)	F^−^*ompT hsdS*_*B*_(${r_{B}}^{-}$${m_{B}}^{-}$) *gal dcm* (DE3) pRARE	Novagen
*E. coli* JM110	F^−^ t*raD*36 *lacI*^q^Δ(lacZ)*M15proA*^+^*B*^+^/*rpsL*^(*StrR*)^ *thr leu thi laY galK galT ara fhuA dam dcm glnV*44 Δ(*lac-proAB*)	ATCC
EEPrbP	BL21 (DE3) carrying p15TV-*prbP*. Amp^r^	Gardner et al., 2016
EEPrbP-N107A	BL21 (DE3) carrying p15TV-*prbP*(N107A). Amp^r^	This work
EEPrbP-G109A	BL21 (DE3) carrying p15TV-*prbP*(G109A). Amp^r^	This work
EEPrbP-I114A	BL21 (DE3) carrying p15TV-*prbP*(I114A). Amp^r^	This work
EEPrbP-R144A	BL21 (DE3) carrying p15TV-*prbP*(R144A). Amp^r^	This work
EEPrbP-R147A	BL21 (DE3) carrying p15TV-*prbP*(R147A). Amp^r^	This work
EEPrbP-E148A	BL21 (DE3) carrying p15TV-*prbP*(E148A). Amp^r^	This work
EERNAPD	BL21 (DE3) carrying p15TV-*rpoD*, pACYCDuet-*rpoB*-*rpoC*, pRSFDuet-*rpoZ*-*rpoA*. Amp^r^. Cm^r^. Kan^r^	This work
ECPRPLK	DH5α carrying pMiniT-*PrplK*. Amp^r^	This work
**PLASMIDS**		
p15TV-L	Expression vector for purification of proteins by nickel affinity chromatography. Amp^r^	Cheryl Arrowsmith, (Addgene plasmid # 26093)
p15TV-*prbP*	*prbP* gene from *L. asiaticus* cloned in the BseRI site of p15TV-L. Amp^r^	Gardner et al., 2016
p15TV-*prbP*(N107A)	p15TV-*prbP* with mutation for N107A. Amp^r^	This work
p15TV-*prbP*(G109A)	p15TV-*prbP* with mutation for G109A. Amp^r^	This work
p15TV-*prbP*(I114A)	p15TV-*prbP* with mutation for I114A. Amp^r^	This work
p15TV-*prbP*(R144A)	p15TV-*prbP* with mutation for R144A. Amp^r^	This work
p15TV-*prbP*(R147A)	p15TV-*prbP* with mutation for R147A. Amp^r^	This work
p15TV-*prbP*(E148A)	p15TV-*prbP* with mutation for E148A. Amp^r^	This work
p15TV-*rpoD*	*rpoD* gene from *L. asiaticus* cloned in the NdeI-BamHI site of p15TV-L. Amp^r^	This work
pACYCDuet-1	Expression vector for co-purification of protein complexes by nickel affinity chromatography. Cm^r^	Novagen
pRSFDuet-1	Expression vector for co-purification of protein complexes by nickel affinity chromatography. Kan^r^	Novagen
pACYCDuet-*rpoB*	*rpoB* gene from *L. asiaticus* cloned in the FseI-PvuI site of pACYCDuet-1 plasmid. Cm^r^	This work
pRSFDuet-*rpoZ*	*rpoZ* gene from *L. asiaticus* cloned in the SalI-NotI site of pRSFDuet-1 plasmid. Kan^r^	This work
pACYCDuet-*rpoB*-*rpoC*	*rpoC* and *rpoB* genes from *L. asiaticus* cloned in the PstI-NotI and FseI-PvuI sites of pACYCDuet-1 plasmid respectively. Cm^r^	This work
pRSFDuet-*rpoZ*-*rpoA*	*rpoA* and *rpoZ* genes from *L. asiaticus* cloned in the EcoRV-KpnI and SalI-NotI sites of pRSFDuet-1 plasmid respectively. Kan^r^	This work
pMiniT	Cloning vector for PCR amplicon	NEB
pMiniT-*PrplK*	Promoter region (−230 to +54 bp) of *rplK* gene cloned into insertion site of pMiniT. Amp^r^	This work

**Table 2:** Primers used in this study.

**Primer**	**Sequence (5′-3′)**
**EMSA**	
EMSA_CLIB_00130_Ext_Fw	CTGTTTTCTTCGAGGTTGGTG
EMSA_CLIB_00130_Ext_Rv	CCGCATTAAACGCCTTACAA
EMSA_CLIB_00130_Fw	CTGATGGTCCGTTTGCTTCT
EMSA_CLIB_00130_Rv_Bio	TGCAGAACCCGACTCTATCTG
**SITE-DIRECTED MUTAGENESIS AND PROTEIN**	
CLIBASIA_01510_LIC-Fw	TTGTATTTCCAGGGCATGACATTCCAACAGAAAAGAGATG
CLIBASIA_01510_LIC-Rv	CAAGCTTCGTCATCA CTATGCGGCTTTATCTTGATTTTC
CLIBASIA_01510_Ext-Fw	GAGTGTGCGTTTGTTTGAAAAG
CLIBASIA_01510_Ext-Rv	CCCACGCGATCTTATCTGAC
CLIB_01510_N107A_FP	CAAGAATACGATGCCAAGATTGCCTCTGGAGACCTAATTGCTATA
CLIB_01510_N107A_RP	TATAGCAATTAGGTCTCCAGAGGCAATCTTGGCATCGTATTCTTG
CLIB_01510_G109A_FP	TACGATGCCAAGATTAACTCTGCAGACCTAATTGCTATAGCGGAA
CLIB_01510_G109A_RP	TTCCGCTATAGCAATTAGGTCTGCAGAGTTAATCTTGGCATCGTA
CLIB_01510_I114A_FP	AACTCTGGAGACCTAATTGCTGCAGCGGAAGTTGTCCGTGACTTA
CLIB_01510_I114A_RP	TAAGTCACGGACAACTTCCGCTGCAGCAATTAGGTCTCCAGAGTT
CLIB_01510_R144A_FP	GCTATATGAATCCGCTCTCAATGCCATGGTCAGAGAAATCGCTGCT
CLIB_01510_R144A_RP	AGCAGCGATTTCTCTGACCATGGCATTGAGAGCGGATTCATATAGC
CLIB_01510_R147A_FP	ATCCGCTCTCAATCGCATGGTCGCAGAAATCGCTGCTGTAAATAGT
CLIB_01510_R147A_RP	ACTATTTACAGCAGCGATTTCTGCGACCATGCGATTGAGAGCGGAT
CLIB_01510_E148A_FP	CGCTCTCAATCGCATGGTCAGAGCAATCGCTGCTGTAAATAGTATC
CLIB_01510_E148A_RP	GATACTATTTACAGCAGCGATTGCTCTGACCATGCGATTGAGAGCG
CLIB_rpoC_Fw_ext	AGCATAACTATTCTGCCAAGACA
CLIB_rpoC_Rv_ext	CGGTGAACCATTTGATCGGC
CLIB_rpoB_Fw_ext	CAAGGATGAGCAACGGGAGAAGGA
CLIB_rpoB_Rv_ext	AGGAATGGGGTGTTTCTGCT
CLIB_rpoA_Fw_ext	ACAATCGGACGCAACTCCTT
CLIB_rpoA_Rv_ext	GCGACGGATGATTGTTCAGC
CLIB_rpoZ_Fw_ext	CATAGAGGTTGGCACCGTCA
CLIB_rpoZ_Rv_ext	TCAACATACATCGAATGCCCCA
CLIB_rpoC_PstI_Fw	TCGCTGCAGATGCAACAAGAGGTCATGAG
CLIB_rpoC_NotI_Rv	ACTGCGGCCGCTTACTCCGCTATACTCCC
CLIB_rpoB_FseI_Fw	GCTGGCCGGCCATGGCAAAAGGCGTTGTGTT
CLIB_rpoB_PvuI_Rv	TGGCGATCGTTACTTTAATTCACATTTAT
CLIB_rpoA_EcoRV_Fw	ATGCTGATATCATGATCCAAAAAAATTGGCAAG
CLIB_rpoA_KpnI_Rv	TCGGGTACCTTAGCACTTATCTTCATATT
CLIB_rpoZ_SalI_Fw	TGTGTCGACATGGCACGTACTACTGTAGA
CLIB_rpoZ_NotI_Rv	ACTGCGGCCGCTCAATCATCTCTCTTATCAG
CLIBASIA_00870_NdeI_Fw	GGCCATATGATGACAATAGGAAA
CLIBASIA_00870_BamHI_Rv	GGATCCCTAACCATCTAAAAAACTC
**SEQUENCING**	
T7	TAATACGACTCACTATAGGG
T7 term	GCTAGTTATTGCTCAGCGG
pMiniT_Fw	ACCTGCCAACCAAAGCGAGAAC
pMiniT_Rv	TCAGGGTTATTGTCTCATGAGCG
ACYCDuetUP1	GGATCTCGACGCTCTCCCT
DuetUP2	TTGTACACGGCCGCATAAT
DuetDOWN1	GATTATGCGGCCGTGTACAA

### Protein purification

Purification of PrbP and its mutants was performed as previously described (Gardner et al., 2016). The 6X His-tagged fusion proteins were over-expressed in *E. coli* BL21 (DE3). Cells were grown in LB broth at 37°C, to OD_600_ = 0.6 and gene expression was induced with 0.5 mM isopropyl-thio-β-D-galactopyranoside (IPTG). Upon induction, the cells were incubated at 17°C for 16 h. The cells were harvested by centrifugation and re-suspended in binding buffer (500 mM NaCl, 5% glycerol, 50 mM HEPES, 5 mM imidazole, pH 7.5). The buffer was amended with Pierce EDTA-free protease inhibitor (Thermo Fisher Scientific). Tris (2-carboxyethyl) phosphine hydrochloride (0.5 mM) was added to the cells immediately before lysing. Cells were lysed by passing through a French pressure cell. The lysates were centrifuged 30 min at 17,000 × g at 4°C and the supernatant was applied to a metal chelate affinity column charged with nickel (His60 Ni Superflow Resin, Clontech). The column was washed extensively with binding buffer containing 25 mM imidazole, and the proteins were eluted from the column in elution buffer (binding buffer with 250 mM imidazole). The purified proteins were dialyzed against 10 mM HEPES (pH 7.5), 500 mM NaCl, 2.5% glycerol, 0.5 mM TCEP, then aliquoted and stored at −80°C. The 6X His-tag of PrbP and its variants was not removed for further experiments.

Purification of the *L. asiaticus* RNAP holoenzyme was performed using a similar protocol, however, the buffers were modified as follow: The binding, wash and elution buffers contained 500 mM NaCl, 5% glycerol, 50 mM Tris-HCl (pH 8) with 5, 15, and 250 mM imidazole, respectively. The purified protein complex was dialyzed against 10 mM Tris-HCl (pH 8), 250 mM NaCl, 50% glycerol, 0.1 mM EDTA, 0.5 mM TCEP, then aliquoted and stored at −80°C. The fused 6X His-tag was not removed for further experiments.

### Electrophoresis mobility shift assays

Electrophoresis mobility shift assays (EMSA) were carried out using the PrbP wild type protein (WT) and PrbP mutants. A fragment of the *rplK* promoter region was amplified by PCR using pre-labeled 5′-biotin primers, and the biotin labeled product was used for the binding target as previously described (Gardner et al., 2016). The electrophoresis mobility shift assay reaction contained 1 ng of 5′-Biotin labeled DNA probe, 10 mM HEPES (pH 7.5), 250 mM NaCl, 5% glycerol, 12.5 ng/μl of non-specific competitor DNA Poly(dI-dC), purified PrbP WT or mutant PrbP protein (0–7 μM), and tolfenamic acid (0–0.5 mM), as indicated. Following incubation at 37°C for 20 min, the samples were analyzed by electrophoresis using 6% acrylamide-bisacrylamide non-denaturing gels, in ice-cold 0.5x Tris-borate EDTA buffer (TBE), pH 8.3. The DNA was then transferred from the polyacrylamide gel to a Hybond-N^+^ membrane (GE Healthcare) by electro-blotting at 250 mA for 45 min, using a semi-dry transfer blot (Fisher Scientific). The transferred DNA was UV-crosslinked and the biotin-labeled DNA was detected using the Phototope-Star Detection Kit (NEB). Membranes were exposed to Kodak X-ray films. Vehicle controls were included in all assays.

### *In vitro* transcription run-off assays

The PrbP proteins used for *in vitro* transcription assays were purified and stored as described above. The recombinant plasmid pMiniT-*PrplK* was linearized using the restriction enzyme NdeI. All proteins were diluted to the working concentration with transcription buffer (40 mM Tris-HCl, pH 8.0, 50 mM KCl, 10 mM MgCl_2_,0.1 mM EDTA). Each 20 μl reaction contained template DNA (5 nM), purified PrbP protein (0–5 μM), and tolfenamic acid (0–0.5 mM), in 1x transcription buffer. The mixtures were pre-incubated for 10 min at 37°C prior to adding the partially purified RNA polymerase. A second incubation was then performed at 37°C for 5 min. The transcription reaction was then initiated by the addition of NTPs (2 mM each of ATP, GTP, and CTP, 1.5 mM of UTP, and 0.5 mM biotin-11-UTP). The reactions were terminated by adding 10 mM EDTA after 30 min of incubation at 37°C. The transcripts were purified, concentrated by ethanol precipitation, and analyzed using 6% acrylamide-bisacrylamide, 7M urea gels, in ice-cold 0.5x TBE buffer, at 100 V for 2.5 h. Transcripts were transferred from the gel to a Hybond-N^+^ membrane (GE Healthcare) by electro-blotting at 380 mA for 40 min in a semi-dry transfer blot (Fisher Scientific). The transferred transcripts were UV-crosslinked and detected using the Phototope-Star Detection Kit (New England Biolabs). The membranes were exposed to Kodak X-ray films to visualize the transcription products. A biotinylated sRNA Ladder (Kerafast) was used as a molecular weight marker.

## Results

### Model of tolfenamic acid binding in PrbP

The structural model of PrbP was generated using SWISS-MODEL server in automated mode. Several templates were identified with coverages >80%, however, all models had low quality scores (GQME <0.6 and negative Z-scores). Although the models were not highly reliable, we have found in other transcription factors that these models can still be utilized as the starting point for *in silico* predictions if followed by biochemical validations (Pagliai et al., 2010; Blancato et al., 2016). Using size exclusion chromatography, it was determined that *L. asiaticus* PrbP is a monomer in solution, with an estimated molecular weight of 24.6 kDa (Supplementary Figure 1A). Since several of the templates retrieved were dimers, those predictions were excluded from further analyses. Among the remaining templates, three models based on the structures of CarD from *Thermus* (PDB# 4L5G, 4XAX, and 4XLR) were found. These CarD structures showed different conformations of the RNA polymerase interacting domain (RID) and DNA binding domain (CTD) (Srivastava et al., 2013; Bae et al., 2015). The distribution of the two functional domains vary from relaxed in the apo structure (PDB#4L5G), to intermediate when in complex with Taq RNAP β-1 lobe (PDB#4XAX), and to constrained in the transcription initiation complex (PDB#4XLR). Since each conformation has been linked to mechanisms of interaction of *Thermus* CarD with DNA and the RNA polymerase (Bae et al., 2015), the models derived from these structures were selected for further studies. The models were named PrbP-MR (using the 4L5G template), PrbP-MI (using the 4XAX template), and PrbP-MC (using the 4XLR template; Figure 1B, Supplementary Figure 2). The modeling input sequence and statistics of the models are listed in Supplementary Table 1.

The docking of tolfenamic acid into PrbP-MI was performed using SwissDock in automated mode, as described in Section Materials and Methods. The best scores were obtained with a pocket formed in the interface of the RID and CTD domains (Supplementary Table 2). In the identified pocket, tolfenamic acid was predicted to interact with residues N107, G109, I114, R144, R147, and E148 (Figure 1C), with several possible orientations. Given the large number of possible docking orientations with no significant difference in energy (<1 kcal/mol difference), the Molecular Docking Server interface was used to narrow down and help determine the docking orientation of tolfenamic acid in the pocket. The most favorable orientation (Figure 1C) had a Gibbs free energy (ΔG) of −6.79 kcal/mol. Residue I114 was predicted to interact with tolfenamic acid atom C1 via hydrophobic interactions (3.4 Å). Residue G109 was predicted to interact with atom Cl1 via halogen bond (3.6 Å). Residue E148 was also predicted to interact with atom Cl1 by halogen bond (3.7 Å) as well as with atoms C6 (2.9 Å). Residue R144 was predicted to interact with atoms C1-C2-C3-C7-C8-C9-C10-C12 (2.1–3.8 Å, respectively). The measurements of distances between the atoms in tolfenamic acid and the amino acids in PrbP are shown in Table 3 and Supplementary Figure 2.

**Table 3:** *In silico* prediction of the interactions between tolfenamic acid and residues within the TaP pocket in PrbP.

**Amino acid**	**Predicted interacting atom(s) on TA**	**Predicted interaction type(s)**	**Measurements of distance [Å]**
N107	NP^*^	NP^*^	3.3
G109	Cl1	Halogen	3.6
I114	C1	Hydrophobic	3.4
R144	C1-C2-C3-C7-C8-C9-C10-C12	Other	2.1-3.8
R147	NP^*^	NP^*^	2.8
E148	Cl1/C6	Halogen/other	3.7/3.9

* *Amino acids within 4 Å radius but the type of interaction has not predicted*.

To verify which amino acids in the predicted TaP pocket are required for interactions with tolfenamic acid, six PrbP mutants were constructed by site-directed mutagenesis, where each of the predicted residues was replaced with alanine. The PrbP WT and mutant constructs were purified with up to 90% purity (Supplementary Figure 1B).

### Mutations in the TaP decrease the inhibitory effect of tolfenamic acid on PrbP/DNA interactions

**Figure 2:**
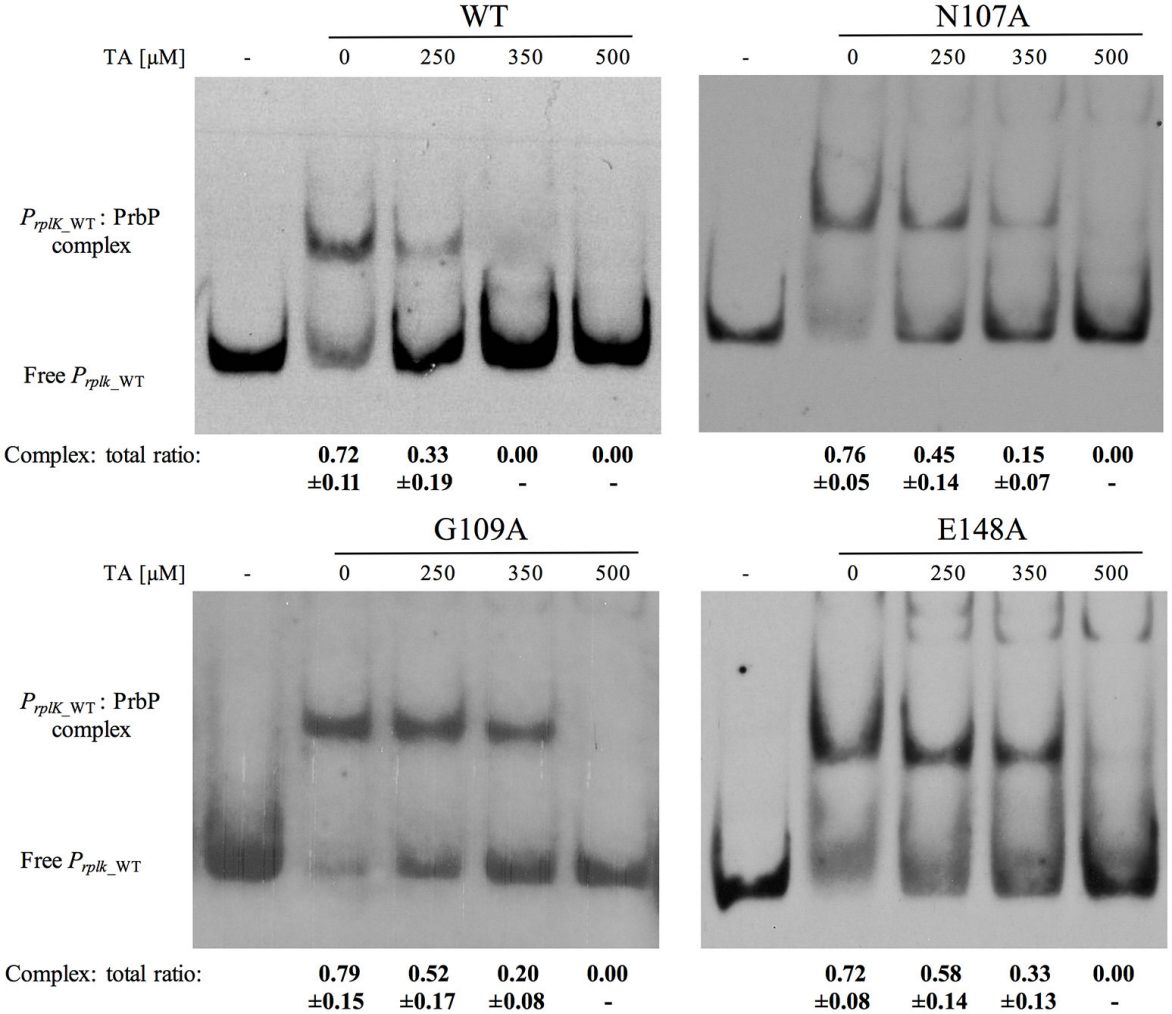
Mutations N107A, G109A, and E148A in TaP decreases the inhibitory effect of tolfenamic acid on PrbP/DNA interactions. The *PrplK* probe and 3.5 μM of each of the PrbP mutants (N107A, G109A, and E148A, as indicated in the top of each panel) were incubated with increasing concentrations (0–500 μM) of tolfenamic acid as indicated. TA, tolfenamic acid; First lane of each panel has no protein added. Ratio was calculated by the band intensity of protein: DNA complex and total DNA. Quantification was based on observations from at least 2 replicates.

The effect of mutations in the TaP pocket, on the ability for PrbP to bind DNA, were first assessed by EMSA, using the known PrbP binding site in the promoter region of *rplK* (*PrplK*) as a probe (Gardner et al., 2016). Of the six mutants tested, no significant difference in DNA binding activity was observed, when compared to the PrbP WT (Supplementary Figure 3). The effect of tolfenamic acid was then examined with each PrbP mutant/*PrplK* complex and compared to the PrbP WT. Complete disruption of the PrbP WT/*PrplK* complex was consistently observed with 350 μM tolfenamic acid, which corresponds to a protein to ligand ratio of 1:100 (Figure 2). Mutations in I114, R144, and R147 showed a similar pattern to the WT, with disruption of the complex also occurring in presence of 350 μM tolfenamic acid (Supplementary Figure 4). Conversely, the presence of 350 μM tolfenamic acid did not completely disrupt the Protein/DNA complex for mutants N107A, G109A, and E148A. The complete disruption of the complex formed with these mutants was only observed at higher concentrations (500 μM) of tolfenamic acid, which corresponds to a protein to ligand ratio of 1:143 (Figure 2). These results indicate that residues N107, G109, and E148 of the predicted TaP pocket, are directly involved in the binding of tolfenamic acid to PrbP.

### Effect of mutations in TaP on PrbP interactions with the RNA polymerase

In our previous report, we showed that PrbP binds specifically to the promoter region of *rplK* and interacts with the β-subunit of the RNA polymerase. Interestingly, the addition of tolfenamic acid significantly decreased expression of *rplK* and 16S ribosomal genes *in vivo*, suggesting a role as a transcriptional activator (Gardner et al., 2016). However, when tested in a two-hybrid system, tolfenamic acid only slightly decreased the interaction between PrbP and RpoB. These results suggest that tolfenamic acid, may inhibit PrbP by disrupting or destabilizing DNA binding.

**Figure 3:**
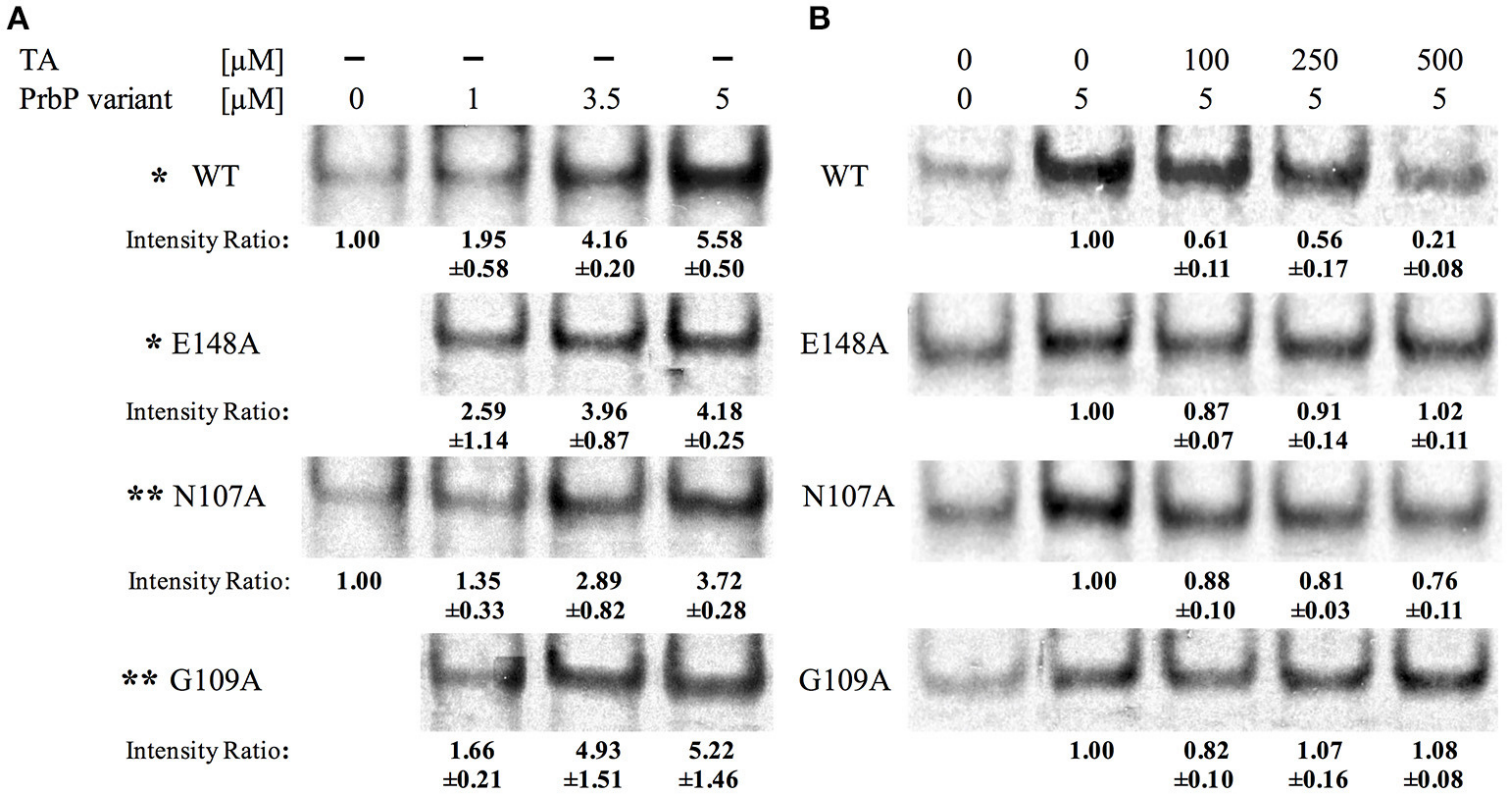
Tolfenamic acid affect PrbP interactions with the RNA polymerase. **(A)** PrbP WT and TaP mutations N107A, G109A, and E148A act as transcriptional activators of *rplK*. Increasing concentrations (0–5 μM) of PrbP WT and mutants were added as indicated; ^*^ and ^**^ indicate that reactions ran on the same gel and share the same control. Ratio was calculated by band intensity normalized to negative control without PrbP or mutants. **(B)** Effect of tolfenamic acid on PrbP WT or mutants (N107A, G109A, and E148A) interactions with the RNA polymerase. PrbP WT, mutants and increasing concentrations of tolfenamic acid (0–500 μM) were added as indicated. Ratio was calculated by band intensity normalized to the control without tolfenamic acid. Quantification was based on observations from at least 2 replicates.

An *in vitro* transcription assay was used to examine the effect of tolfenamic acid on PrbP, and its interactions with the RNA polymerase during transcription initiation. All five components of the *L. asiaticus* RNA polymerase (αββ′ω), and the sigma 70 factor, were cloned into a *E. coli* co-expression system, and purified by affinity chromatography (Supplementary Figure 5). The partially purified RNA polymerase was used for *in vitro* transcription run-off assays, with plasmid DNA carrying *PrplK* as a template. When added to the assay reaction, PrbP significantly increased the transcript concentration in a dose dependent manner, with the highest increase in transcription observed with 5 μM PrbP (Figure 3A). These results confirmed that PrbP WT acts as a transcriptional activator for *rplK*. These findings are consistent with previous studies on CarD from *Mycobacterium*, where CarD was found to increase transcriptional activity of the *Mycobacterium* RNAP, while it had no effect on transcription when added to the *E. coli* RNAP (Davis et al., 2015; Rammohan et al., 2015). The transcription of *PrplK* was then examined in presence of the TaP mutants, where mutants E148A, N107A, and G109A were all found to behave similar to the WT (Figure 3A).

*In vitro* transcription was also used to examine the effect of increasing concentrations of tolfenamic acid, on the transcription of *rplK*. The addition of 250 μM tolfenamic acid was found to decrease *rplK* transcription by 44% with the PrbP WT, and at higher concentrations (500 μM tolfenamic acid) a 79% decrease in transcription was observed (Figure 3B). No significant decrease in *rplK* transcription was observed with mutants G109A and E148A. With the N107A mutant, the addition of tolfenamic acid was also found to decrease the transcription of *rplK*, but to a much lesser extent (19 and 24% with 250 and 500 μM tolfenamic acid, respectively; Figure 3B). The reduced sensitivity to tolfenamic acid observed with PrbP mutants N107A, G109A, and E148A supports an important role of these residues for a ligand inhibition of the PrbP activity. These results also suggest that residues G109 and E148 are more significant for interactions with tolfenamic acid than residue N107.

### Residues in the TaP are conserved in the CarD_CdnL_TRCF family

**Figure 4:**
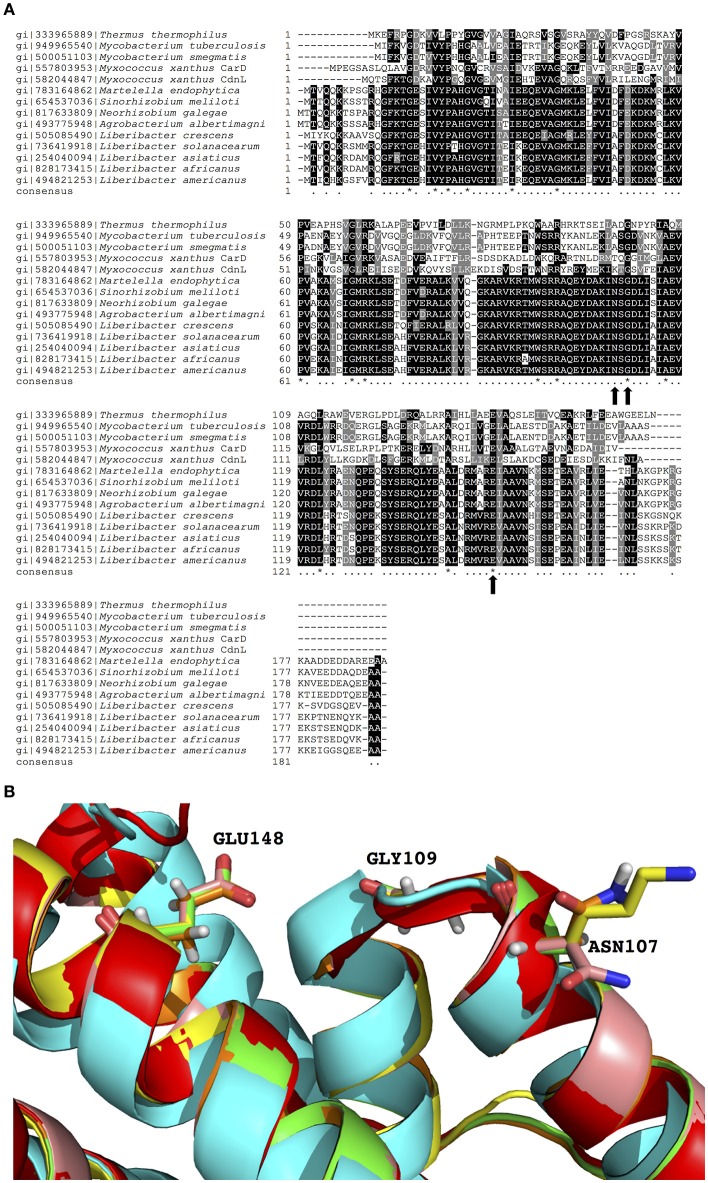
Sequence and structural alignments of PrbP and CarD_CdnL_TRCF family members. **(A)** Multiple sequence alignment of PrbP and PrbP homologs of the CarD_CdnL_TRCF family. Sequences were extracted from NCBI database and multiple alignments were performed using Clustal Omega. Black dots, 60% and up conserved; asterisk, completely conserved. **(B)** Structural alignment of PrbP-MI and PrbP homologs. The homologs from *S. meliloti* (pink), *M. smegmatis* (green), *M. xanthus* (yellow), were modeled using PDB# 4XAX as the template. The crystal structures used are from *T. thermophilus* (PDB# 4L5G, red, Srivastava et al., 2013), *M. tuberculosis* (PDB#4KBM, blue, Gulten and Sacchettini, 2013).

To explore the potential significance of TaP in the CarD_CdnL_TRCF family, multiple linear sequence alignments and structural alignments were performed. In this analysis, we included the sequence of each PrbP homolog with structural data available (*Thermus, Mycobacterium*, and *Myxococcus*) as well as members of the Rhizobiaceae family. It was found that residues G109 and E148 are conserved in all members of the CarD_CdnL_TRCF family that were analyzed, while N107 is conserved in the Rhizobiaceae family (Figures 4A,B). The conservation of these residues in the ligand binding pocket suggests that they may be functionally relevant for members of the CarD_CdnL_TRCF family, and that interactions with these proteins may be modulated by an unknown native ligand.

## Discussion

CarD_CdnL_TRCF is an expanding family of proteins with an atypical DNA binding domain. Members of this family are widely distributed and have been identified in several human and plant pathogens, including *Mycobacterium, Clostridium, Bacillus*, and *Liberibacter* species. Initial studies in *Myxococcus xanthus* identified the role of this family as transcriptional regulators. Additional studies in *Myxococcus, Mycobacterium*, and *Thermus species* later revealed that members of this family are important RNA polymerase interacting proteins, that regulate transcription initiation (Stallings et al., 2009; Garcia-Moreno et al., 2010; Bae et al., 2015; Rammohan et al., 2015). In some genera, they are essential proteins (Stallings et al., 2009; Garcia-Moreno et al., 2010), suggesting their potential use as therapeutic targets for new antimicrobial compounds.

PrbP is a newly characterized member of the CarD_CdnL_TRCF family from *L. asiaticus* (Gardner et al., 2016). In our previous study, we found that PrbP interacts with RNAP, however, contrary to previous reports of homologs, *L. asiaticus* PrbP binds specifically to the *rplK* promoter region, and the 16S ribosomal gene (Gulten and Sacchettini, 2013; Landick et al., 2014; Gardner et al., 2016). Tolfenamic acid is a small molecule that was found to disrupt PrbP/DNA interactions *in vitro*. It was also found to be an effective inhibitor of PrbP *in vivo* (using HLB-infected citrus seedlings), where the overall transcriptional activity of *L. asiaticus* was observed to decrease after treatment with tolfenamic acid.

To further examine the effect of tolfenamic acid on PrbP/RNAP interactions, as well as to identify the tolfenamic acid binding pocket in PrbP, we performed *in vitro* transcription assays. Purification of a bacterial RNA polymerase is usually accomplished by tagging one subunit of the polymerase, followed by affinity chromatography to pull-down the entire complex in the target bacteria (Murakami et al., 1997; China and Nagaraja, 2010; Svetlov and Artsimovitch, 2015). Due to the inability to culture *L. asiaticus* under standard laboratory conditions, an alternative purification strategy was employed. To this end, all five components of the *L. asiaticus* RNA polymerase (αββ′ω), and the sigma 70 factor, were cloned into a *E. coli* co-expression system, and purified (Supplementary Figure 5). In *Mycobacterium*, the RNAP/open promoter complexes are highly unstable, however, previous studies have shown that PrbP homologs can effectively stabilize mycobacterial RNAP/open promoter complexes, resulting in increased transcriptional activity. Conversely, the open promoter complexes formed by *E. coli* RNAP are highly stable, and transcriptional activity is unaffected by the presence of PrbP homologs (Davis et al., 2015; Rammohan et al., 2015). Based on these findings, the partially purified *L. asiaticus* RNAP was used to perform *in vitro* transcription assays, as any potential *E. coli* RNAP contaminants should not significantly affect the results of the assay. Similar to the results reported in *Mycobacterium* for CarD, the addition of PrbP increased the transcription of the *rplK* promoter by the *L. asiaticus* RNA polymerase. One observation of the inhibitory effect of tolfenamic acid is that higher concentrations (500 μM) did not completely abolish the activity of PrbP *in vitro*, however, lower concentrations (70 μM) of tolfenamic acid were previously found to inhibit *L. crescens* growth and affect the transcriptional activity of *L. asiaticus in vivo* (Gardner et al., 2016). We hypothesize that even a small decrease in PrbP activity could significantly affect cell viability due to the slow duplication rate of *L. crescens* (24 h, Fagen et al., 2014). It is also possible that the inhibitory effect of tolfenamic acid on PrbP would affect the expression of other genes that may be essential for growth.

To investigate the inhibition mechanism of PrbP, we tested the possibility that tolfenamic acid could block PrbP binding to RNAP using a *E. coli* two-hybrid system, and only observed a 15% decrease in the protein-protein interactions (Gardner et al., 2016). Since the low level of inhibition was not sufficient to explain the significant decrease in transcriptional activity observed *in vivo* (Gardner et al., 2016) and *in vitro*, the results suggest the mechanism of tolfenamic acid inhibition is unlikely to be the direct binding to the PrbP/RNAP interface. In this report, we used *in silico* structure modeling and docking to identify TaP as the tolfenamic acid binding pocket in PrbP. Interestingly, TaP is located at the interface of the RNAP and DNA binding domains. Recent literature on CarD from *T. thermophiles*, where it was suggested that the RID and CTD of CarD are flexible in solution, and that CarD only assumes its active conformation when the CTD is positioned to interact with the promoter DNA (Bae et al., 2015). Based on our findings, and the structural data available, we hypothesize that preventing the formation of the PrbP active conformation is the basis for the inhibition mechanism of tolfenamic acid.

*Thermus* CarD structures PDB# 4L5G (apo structure) (Srivastava et al., 2013), PDB# 4XAX (in complex with RNAP β-1 lobe) and PDB# 4XLR (with the transcription initiation complex) (Bae et al., 2015) showed that the CTD and RID functional domains of CarD assume different conformations depending on the environment (i.e., presence of RNAP components or transcription initiation complex). These conformations seem to be relaxed (PDB# 4L5G), intermediate (PDB# 4XAX) or constrained (PDB# 4XLR). Using these structures as templates, the structural modeling of PrbP showed that the TaP is only formed in the intermediate conformation (PrbP-MI). Using *in silico* docking, we tested if tolfenamic acid would bind to the same location if PrbP was modeled using the CarD apo structure from *T. thermophilus* (PDB# 4L5G) or the structure obtained from CarD crystallized with the transcription initiation complex (PDB# 4XLR). When PrbP was modeled to 4L5G, tolfenamic acid was positioned further away from the interacting amino acids N107, G109, and E148 (average distance 4.3–5.6 Å, Supplementary Figure 2C) when compared to the docking model based on PDB# 4XAX (average distance 3.3–3.9 Å, Supplementary Figure 2A), indicating that the relaxed distribution may not aid in tolfenamic acid binding. Structural alignment of the PrbP-MC model and the tolfenamic acid/PrbP-MI docking prediction, revealed that the tolfenamic acid molecule would clash with amino acids R144 and R147 (Supplementary Figure 2D). These *in silico* predictions indicate that PrbP may be unable to assume a constrained (“active”) conformation when tolfenamic acid binds to the predicted pocket, hence, inhibiting the transcription activation activity of PrbP.

All together, these *in silico* analyses suggest that the formation of TaP in PrbP may be dependent on the RID and CTD domain conformation. The conserved nature of the critical amino acids in TaP suggests that the identified binding pocket may also be a critical component for regulating the activity of members of the CarD_CdnL_TRCF family, through interactions with native small molecules. Previous reports on members of the CarD_CdnL_TRCF family have focused on the identification of residues that mediate binding to the promoter DNA, as well as those that mediate interactions with the β1-lobe of the RNAP. The effect of small molecules that modulate these interactions, however, has not been addressed. In *L. asiaticus*, the expression of *prbP* was found to remain stable following chemical inactivation of PrbP/DNA interactions, indicating that *prbP* expression is not auto-regulated at the level of transcription (Gardner et al., 2016). Based on the constitutive nature of PrbP in *L. asiaticus*, and the finding that tolfenamic acid binding inhibits the activity of PrbP, we hypothesize that the activity of PrbP is modulated by the binding of an unknown signaling molecule which may maintain PrbP in an “inactive” conformation until its activity is required, however, further studies are required to identify the native molecule.

In mycobacteria, the expression of *carD* is highly induced in response to DNA damage, oxidative stress, and starvation conditions (Stallings et al., 2009). Conversely, the rRNA levels in wild-type *M. smegmatis* are repressed during starvation and oxidative stress, however, the repression was abolished when CarD was depleted *in vivo*. Microarray and qRT-PCR experiments confirmed that CarD depletion resulted in transcriptional upregulation of genes encoding ribosomal proteins and translation factors (Stallings et al., 2009). These observations indicate that in mycobacteria, CarD is involved in stringent response. One well-known bacterial stringent control model is the DksA-(p)ppGpp in *E. coli* (Paul et al., 2004, 2005; Perederina et al., 2004; Potrykus and Cashel, 2008; Atkinson et al., 2011; Lennon et al., 2012; Ross et al., 2013, 2016), where ppGpp is a signaling molecule synthesized during amino acid starvation, that works in conjunction with DksA to regulate transcription. It is proposed that DksA forms a synergetic binding with ppGpp at the RNA polymerase secondary channel rim, while stabilizing ppGpp binding to a second site 60 Å away, resulting in a modified transcription initiation complex (Ross et al., 2013, 2016). In *M. tuberculosis* and *M. smegmatis*, which lack DksA, CarD is required for ppGpp to elicit a stringent response (Stallings et al., 2009). Although, investigation of CarD-ppGpp interaction is yet to be done, the fact that mycobacteria CarD can functionally complement an *E. coli* Δ*dksA* strain (Stallings et al., 2009) suggests that ppGpp may serve as a native ligand for some members of the CarD_CdnL_TRCF family. Similar to *M. tuberculosis* and *M. smegmatis, L. asiaticus* also lacks DksA, suggesting PrbP may be involved in stringent response as well. In *L. asiaticus*, the signaling molecule is unlikely to be ppGpp, as the enzymes required for its synthesis are absent in the genome, however, a similar regulatory mechanism may exist.

In summary, we identified a conserved ligand binding pocket (TaP), that is involved in modulating the DNA binding activity of PrbP *in vitro*, and may also serve as the binding site for a native signaling molecule to regulate PrbP activity *in vivo*. Taken together, these results indicate that members of the CarD_CdnL_TRCF family might be regulated through interactions with small molecules during stress response or during interactions with the host.

## Author contributions

LP, FP, and GL designed and analyzed the experiments. LP, CLG, and FP performed experiments. CLG and CFG provided assistance in experiments, contributed in the analysis of results and preparation of the manuscript. LP and GL wrote the paper. GL conceived and coordinated the study.

### Conflict of interest statement

A patent application has been submitted for the use of tolfenamic acid for the treatment of HLB. The authors declare that the research was conducted in the absence of any commercial or financial relationships that could be construed as a potential conflict of interest.

## Abbreviations

PrbP: Predicted RNA polymerase binding protein
HLB: Huanglongbing
RNAP: RNA polymerase
EMSA: electrophoretic mobility shift assay
TaP: tolfenamic acid pocket
PrplK: rplK promoter region.
