# Supplementary material for: Identification of the Tolfenamic Acid Binding Pocket in PrbP from Liberibacter asiaticus
Source: Front Microbiol. 2017 Aug 23;8:1591. doi: 10.3389/fmicb.2017.01591 (PMC5572369; doi:10.3389/fmicb.2017.01591)
Supplement: Supplementary file 1 [file DataSheet1.DOCX]

**Supplementary Figure 1. (A) Size exclusion chromatography of PrbP.** The PrbP protein fused with 6X His-tag was purified as described in Materials and Methods. Samples were passed through Superose-12 10/300 GL column equilibrated with 50 mM Tris pH=8.0, 500 mM NaCl, 2.5% glycerol and 0.5 mM DTT. Flow rate was 0.5 mL/min. The void volume of the column was determined using Blue dextran 2000. The observed peaks were labeled based on standard curve determined by a combination of protein molecular weight standards, including IgG (150 kDa), BSA (66 kDa), Albumin (45 kDa), Trypsinogen (24 kDa), Cytochrome C (12.4 kDa), and Vitamin B12 (1.36 kDa), passed through the column under the same conditions. (**B) SDS-PAGE of the Purification of *L. asiaticus* PrbP and its mutants.** Lane 1, Molecular weight markers; Lane 2, PrbP WT; Lane 3, PrbP N107A; Lane 4, PrbP G109A; Lane 5, PrbP I114A; Lane 6, PrbP R144A; Lane 7, PrbP R147A; Lane 8, PrbP E148A. Each lane contains 2 µg of protein.

**Supplementary Figure 2. Graphical representation of the distance between the interacting amino acids in PrbP models and the tolfenamic acid atoms.** **(A) and (B)** The PrbP-MI model using *T. thermophilus* CarD co-crystalized with RNA polymerase β-1 lobe (PDB ID :4XAX, Bae et al., 2015) as template. Orange, cartoon representation of the model PrbP-MI; blue sticks, tolfenamic acid; orange sticks, predicted interacting amino acids in TaP. **(C)** The PrbP-MR model using *T. thermophilus* CarD (PDB ID: 4L5G, Srivastava et al., 2013) as template, was superimposed on PrbP-MI with docked ligand. Green, cartoon representation of the PrbP-MR; blue sticks, tolfenamic acid; green sticks, predicted interacting amino acids in TaP. **(D)** The PrbP-MC model using *T. aquaticus* CarD crystallized with transcription initiation complex (PDB ID: 4XLR, Bae et al., 2015) as template, was superimposed on PrbP-MI docked with ligand. Red, cartoon representation of the PrbP-MC; blue sticks, tolfenamic acid; red sticks, predicted interacting amino acids in TaP. The measurements of distances between ligand and predicted amino acids are shown as dashed black lines.

**Supplementary Figure 3. Mutations in the TaP pocket do not significantly affect DNA binding activity.** Titration of the *Prplk* probe and increasing concentrations (0-5µM) of PrbP WT and the mutants N107A, G109A, E148A, R147A, R144A and I114A, as indicated on top of each panel; no protein was added to the first lane of each panel.


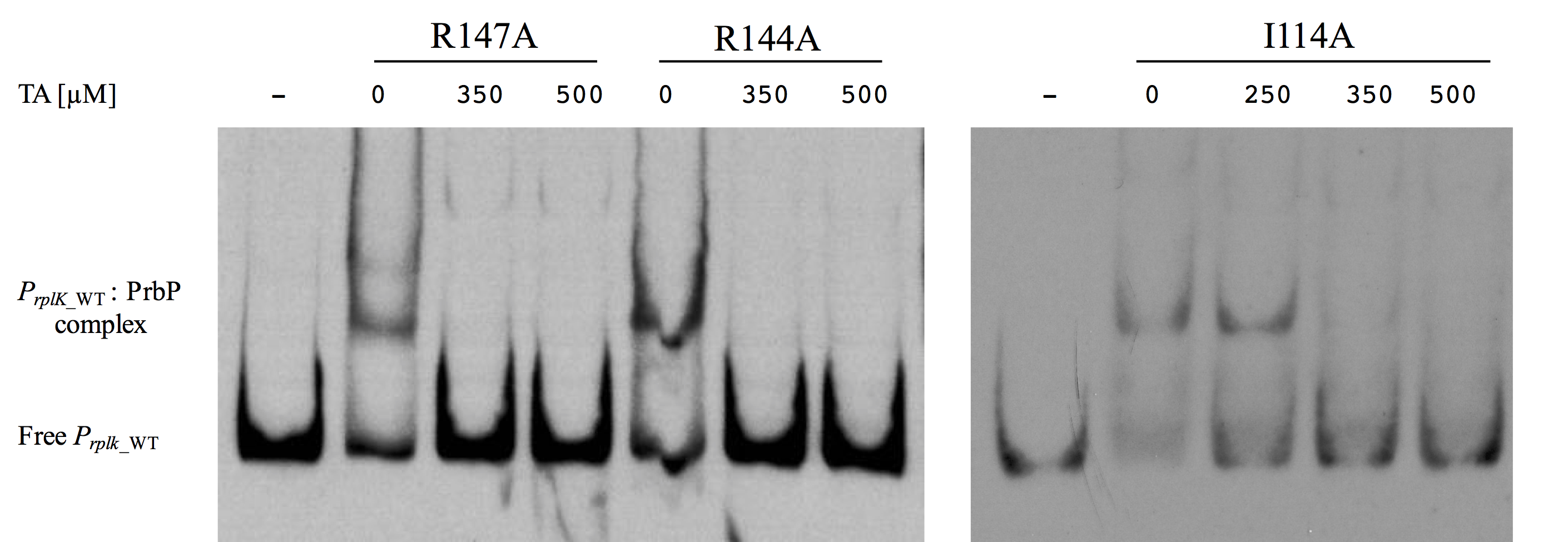


**Supplementary Figure 4. Mutations I114A, R144A and R147A in the TaP pocket do not affect the inhibitory effect of tolfenamic acid on PrbP/DNA interactions.** The *Prplk* DNA probe and 3.5 μM of each of the TaP mutants R144A, R147A and I114A, were incubated with increasing concentrations (0-500 µM) of tolfenamic acid as indicated on top of each panel. TA, tolfenamic acid; no protein was added to the first lane of each panel.


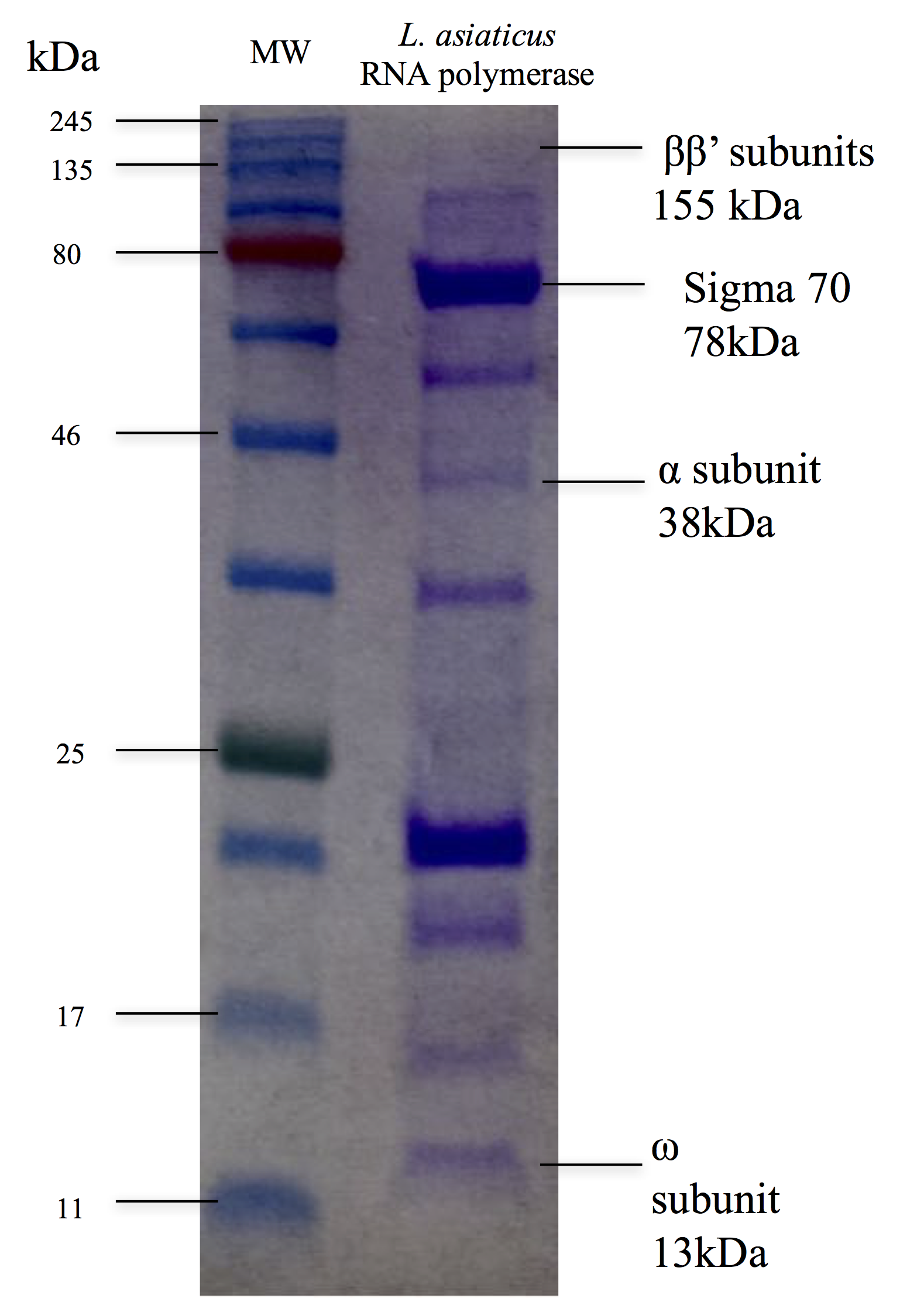


**Supplementary Figure 5. Partial purification of *in vivo* assembled *L. asiaticus* RNA polymerase holoenzyme**. SDS-PAGE of the purified fraction of *L. asiaticus* RNA polymerase holoenzyme (~35% purity) stained by coomassie blue. Color pre-stained protein standard (NEB) was used to estimate the molecular weight. Subunits of the RNA polymerase and sigma factor 70 are indicated with its predicted molecular weight. The purity of the polymerase was calculated based on the quantification of the individual components and divided by the intensity of the total protein content using ImageJ.

**Supplementary Table 1. Modeling input sequence and statistics of the PrbP models.**

| **Input sequence:** MTFQQKRDAMRQGFRTGEHIVYPAHGVGTITEIKEQEVAGMKLEFFVIAFDKDKMCLKVPVGKAIDIGMRKLSEAHFVERALKLVRGKARVKRTMWSRRAQEYDAKINSGDLIAIAEVVRDLHRTDSQPEKSYSERQLYESALNRMVREIAAVNSISEPEAINLIEVNLSSKSSKTEKSTSENQDKAA | | | | | | | | |
| --- | --- | --- | --- | --- | --- | --- | --- | --- |
| **Model** | **Template PDB#** | **Description** | **Coverage (%)** | **Sequence Identity (%)** | **Oligo status*** | **GMQE** | **QMEAN** | **Reference** |
| PrbP-MR | 4L5G | *Thermus* CarD | 81 | 24.18 | monomer | 0.55 | -1.25 | (Srivastava et al., 2013) |
| PrbP-MI | 4XAX | *Thermus* CarD: Taq RNAP β1 lobe | 84 | 23.42 | monomer | 0.57 | -2.03 | (Bae et al., 2015) |
| PrbP-MC | 4XLR | *Thermus* CarD: Taq RPo | 85 | 23.27 | monomer | 0.57 | -2.57 | (Bae et al., 2015) |
| PrbP-4XLS | 4XLS | *Thermus* CarD: Taq RPo | 85 | 23.27 | monomer | 0.58 | -1.88 | (Bae et al., 2015) |
| PrbP-2LWJ | 2LWJ | *Myxococcus* CdnL | 80 | 32.45 | monomer | 0.56 | -4.16 | (Gallego-García et al., 2014) |
| PrbP-4KBM | 4KBM | *Mycobacterium* CarD: RNAP β1- β2 domains | 81 | 28.10 | monomer | 0.54 | -0.79 | (Gulten and Sacchettini, 2013) |
| PrbP-4ILU | 4ILU | *Mycobacterium* CarD | 85 | 27.50 | dimer | 0.59 | -2.29 | (Kaur et al., 2014) |
| PrbP-4MFR | 4MFR | *Mycobacterium* CarD | 85 | 27.50 | dimer | 0.58 | -2.24 | (Kaur et al., 2014) |

* Oligo-status of PrbP homologs in the structures.

**Supplementary Table 2. Results obtained from the *in silico* docking of tolfenamic acid to PrbP-MI*.**

| **Ligand cluster#** | **Orientations in cluster** | **Location** | **Estimated ∆G (kcal/mol)** | **Full fitness (kcal/mol)** |
| --- | --- | --- | --- | --- |
| 0 | 29 | RID and CTD interface | -7.30 to -5.80 | -1165.91 to -1143.71 |
| 1 | 25 | RID and CTD interface | -7.30 to -3.17 | -1164.81 to -1074.99 |
| 2 | 10 | RID and CTD interface | -7.04 to -6.71 | -1163.47 to -1161.43 |
| 3 | 11 | RID and CTD interface | -7.30 to -6.47 | -1163.26 to -1138.57 |
| 4 | 8 | RID and CTD interface | -7.12 to -6.33 | -1163.195 to -1147.36 |
| 5 | 13 | RID and CTD interface | -6.84 to -6.22 | -1160.46 to -1143.92 |
| 6 | 3 | RID and CTD interface | -6.50 to -6.48 | -1160.36 to -1159.48 |
| 7 | 12 | RID and CTD interface | -7.04 to -6.33 | -1160.20 to -1145.99 |
| 8 | 8 | RID and CTD interface | -6.65 to -6.60 | -1159.97 to -1149.50 |
| 9 | 5 | RID and CTD interface | -6.38 to -5.41 | -1159.75 to -1129.79 |
| 10 | 16 | RID and CTD interface | -6.78 to -4.62 | -1159.74 to--1093.13 |
| 13 | 3 | CTD domain | -6.52 to -6.50 | -1159.01 to -1158.88 |
| 25 | 8 | CTD domain | -5.43 to -5.42 | -1155.79 to -1155.67 |
| 27 | 5 | CTD domain | -6.35 to -6.00 | -1154.51 to -1150.51 |
| 18 | 6 | RID and CTD loop | -7.18 to -6.77 | -1151.81 to -1157.75 |
| 38 | 1 | RID and CTD loop | -6.28 | -1146.15 |

* Data shown in this table contain the first 10 ligand clusters located at the RID and CTD interface and all ligand clusters located at CTD domain and RID and CTD loop.
